# SARS-CoV-2: phenotype, genotype, and characterization of different variants

**DOI:** 10.1186/s11658-022-00352-6

**Published:** 2022-06-17

**Authors:** Mohammadreza Saberiyan, Elham Karimi, Zahra Khademi, Parvaneh Movahhed, Amir Safi, Ameneh Mehri-Ghahfarrokhi

**Affiliations:** 1 Cellular and Molecular Research Center, Basic Health Sciences Institute, Shahrekord University of Medical Sciences, Shahrekord, Iran; 2 Department of Medical Genetics, School of Medicine, Hormozgan University of Medical Sciences, Bandar Abbas, Iran; 3 Department of Genetics, Faculty of Basic Sciences, Shahrekord University, Shahrekord, Iran; 4 Department of Medical Laboratory Sciences, School of Allied Medical Sciences, Shahid Beheshti University of Medical Sciences, Tehran, Iran; 5 Clinical Biochemistry Research Center, Basic Health Sciences Institute, Shahrekord University of Medical Sciences, Shahrekord, Iran; 6 Clinical Research Development Unit, Hajar Hospital, Shahrekord University of Medical Sciences, Shahrekord, Iran

**Keywords:** SARS-CoV-2 variants, Omicron, COVID-19, Vaccine

## Abstract

Severe acute respiratory syndrome coronavirus 2 (SARS-CoV-2) is the cause of coronavirus disease 2019 (COVID-19), a major international public health concern. Because of very similar amino acid sequences of the seven domain names, SARS-CoV-2 belongs to the Coronavirinae subfamily of the family Coronaviridae, order Nidovirales, and realm Riboviria, placed in exceptional clusters, but categorized as a SARS-like species. As the RNA virus family with the longest genome, the Coronaviridae genome consists of a single strand of positive RNA (25–32 kb in length). Four major structural proteins of this genome include the spike (S), membrane (M), envelope (E), and the nucleocapsid (N) protein, all of which are encoded within the 3′ end of the genome. By engaging with its receptor, angiotensin-converting enzyme 2 (ACE2), SARS-CoV-2 infects host cells. According to the most recent epidemiological data, as the illness spread globally, several genetic variations of SARS-CoV-2 appeared quickly, with the World Health Organization (WHO) naming 11 of them. Among these, seven SARS-CoV-2 subtypes have received the most attention. Alpha (B.1.1.7), Beta (B.1.351), Gamma (P.1), Delta (B.1.617.2), and Omicron (B.1.617.2) are now designated as variations of concern (VOC) (B.1.1.529). Lambda (C.37) and Mu are variations of interest (VOI) (B.1.621). The remaining six are either being monitored or are no longer considered a threat. On the basis of studies done so far, antiviral drugs, antibiotics, glucocorticoids, recombinant intravenous immunoglobulin, plasma therapy, and IFN-α2b have been used to treat patients. Moreover, full vaccination is associated with lower infection and helps prevent transmission, but the risk of infection cannot be eliminated completely in vaccinated people.

## Introduction

An unusual viral pneumonia was first reported in Wuhan, China in December 2019, and the emergence of a new coronavirus was later confirmed after sequencing the genomic structure of the virus isolated from airway epithelial cells of patients carrying the viral disease, which was subsequently given the official name, COVID-19 [1]. On the basis of its similarities to severe acute respiratory syndrome (SARS-CoV), the new virus was named SARS-CoV-2 [2]. Coronaviruses can cause diseases in humans and vertebrates [3]. NL63, HKU1, OC43, and 229E CoVs do not cause severe symptoms in humans, while SARS-CoV and Middle East Respiratory syndrome coronavirus (MERS-CoV) are zoonotic in origin and cause severe disease and sometimes even fatal diseases [4]. Between 2002 and 2003, SARS-CoV, which originated in China’s Guangdong Province, resulted in 8000 clinical cases worldwide, of which 10% resulted in death [5–7]. Since 2012, MERS-CoV has caused persistent epidemics and is the pathogen responsible for the outbreak of severe respiratory disease in Saudi Arabia [8]. SARS-CoV-2 is known to belong to the genus *Betacoronavirus*, and its genome sequence is similar to SARS-CoV, which targets ACE2 to enter the cell [9]. Preliminary studies have shown that, in patients with SARS-CoV-2, various organs, including the lungs, liver, kidneys, gastrointestinal tract, and heart, are affected [10]. An important point to keep in mind is that most of the injuries in patients infected with SARS-CoV-2 are caused by activation of the inflammatory system followed by a cytokine storm, which can lead to damage to various organs [11]. Various mutations in the virus genome have formed new strains of the virus. The World Health Organization (WHO) has identified Alpha, Beta, Delta, Gamma, and Omicron as variants of concern (VOC) and Lambda and Mu as latest variants of interest (VOI) (updated February 2022)[12]. Although these strains have the same origin, the transmissibility, severity of disease, drug efficacy, vaccine efficacy, and their pathogenesis are different. In this study, we intend to review the genotype, phenotype, and pathogenesis of different strains of SARS-CoV-2.

## The taxonomy of SARS-CoV-2

**Fig. 1:**
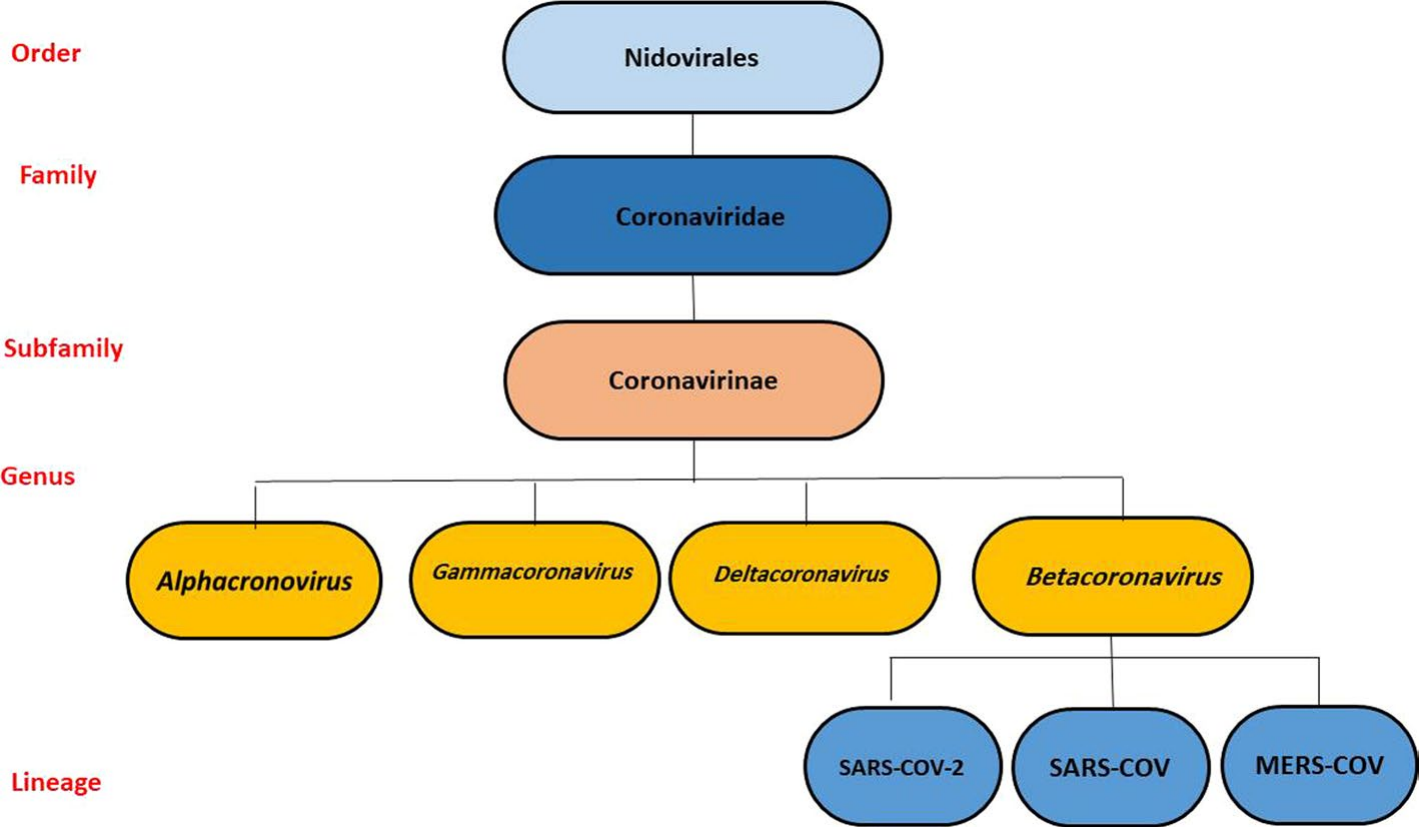
Phylogenetic form: phylogenetic analysis of SARS-CoV-2 and *Betacoronavirus* genomes of other viruses in the Orthocoronavirinae subfamily [1]

The Coronaviridae family belongs to the Cornidovirineae suborder, Nidovirales order, and realm Riboviria, which has Letovirinae, Orthocoronavirinae, and Pitovirinae subfamilies. The Coronavirinae subfamily includes four genera: *Alphacoronovirus* (α-CoV), *Betacoronavirus* (β-CoV), *Deltacoronavirus* (δ-CoV), and *Gammacoronavirus* (γ-CoV) [13]. Phylogenetic relationships form a clade in the subgenus *Sarbecovirus*, subfamily Orthocoronavirinae (Fig. 1). Evolutionary analysis of coronaviruses has shown that α-CoV and β-CoV originate from bats and rodents, while γ-CoV and δ-CoV originate from bird species [14]. Further results of viral genome sequencing, along with other reports, indicate that the SARS-CoV-2 virus is 75–80% identical to SARS-CoV and even more closely associated with different bat coronaviruses [15].

## General features (genotypes and phenotypes)

The size of the virions is 118–140 nm. Nucleocapsids in the subfamily Coronavirinae are flexible. The genome of these viruses consists of single-strand positive-sense RNA with a length of 25–32 kb and is capped and polyadenylated with 38% GC content. The surface of the virions is covered with spikes. Genome transcription and replication are cytoplasmic. Genome-length RNA acts as the mRNA for a long polyprotein precursor that encodes several nonstructural proteins (nsps) including RNA-dependent RNA polymerase (RdRp). The high rate of pattern change is due to the discontinuous transcription seen in these viruses [16, 17].

**Fig. 2:**
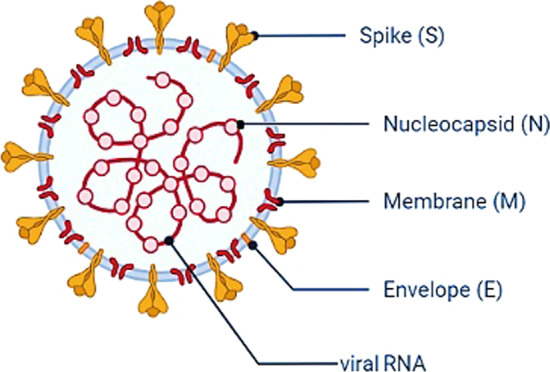
Coronavirinae subfamily structure. Coronavirinae subfamily contains surface viral proteins, namely spike glycoprotein (S), which mediates interaction with the ACE2 cell surface receptor. Viral membrane glycoproteins (M) and envelope (E) of SARS-CoV-2 embedded in the lipid bilayer of viral membranes coat the viral RNA helical nucleocapsid [16] (created with BioRender.com)

**Fig. 3:**
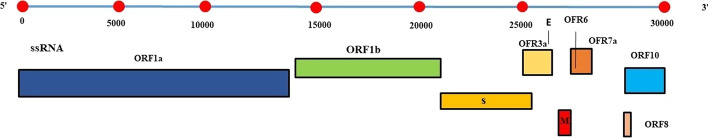
SARS-CoV-2 genome organization. SARS-CoV-2 genome contains 6–12 open reading frames (ORFs) with 5′ and 3′ flanking untranslated regions (UTRs) [17]

SARS-CoV-2 genomic structure includes 5′-leader sequence ORF1/ab-S-ORF3a-E-M-ORF6a-ORF7a-ORF7b-ORF8-N-ORF10-3′ from 5′ to 3′, respectively, in which some transcription regulatory sequences (TRS) are detected at the junction between each open reading frame (ORF). Also, the hemagglutinin-esterase gene that is recognized in other β-CoVs is not detected in SARS-CoV-2 [18, 19]. SARS-CoV-2 contains surface indicators, namely spike glycoprotein (S), which mediates connection with the ACE2 receptor. Viral membrane glycoproteins (M) and envelope (E) of SARS-CoV-2 located in the lipid bilayer of viral membranes coat the viral RNA helical nucleocapsid (Fig. 2) [16]. The 25–32 kb positive-strand RNA genome contains 6–12 ORFs with 5′ and 3′ flanking untranslated regions (UTRs) (Fig. 3). Nonstructural proteins (nsps) are required for genome replication and transcription; they are encoded by about two-thirds of the genome. Sequence variability between SARS-CoV-2 and SARS-CoV did not show notable differences in nsps and ORFs. nsps contains viral cysteine proteases including papain-like protease (nsp3), chymotrypsin-like protease, 3C-like, or primary protease (nsp5), RNA-dependent RNA polymerase (nsp12), helicase (nsp13), and others possibly transcribed that are required for SARS-CoV-2 transcription and genome replication [17]. In addition to nsps, the four general structural components are spike surface glycoprotein (S), membrane nucleocapsid protein (N), envelope (E), and other proteins encoded by ORFs. Studies have shown that a variety of mutations, including missense, synonymous, insertion, deletion, and noncoding mutations, cause changes in the corona genome, with missense and synonymous mutations being the most common. Among them, *nsp1*, *nsp2, nsp3*, *nsp12*, and *nsp15* of ORF1ab, *S*, and *ORF8* genes were recognized to have considerably more changes, although so far no mutations have been identified on the *M* gene [20].

## Cellular infection

The process of coronavirus entering the host cell begins with the binding of spike protein to the membrane receptor and the cleavage of S protein by host enzymes. The role of ACE2, NRP-1, CD147, and P2X7 receptor and TMPRSS2 serine proteases in the entry of SARS-CoV-2 into the host cell has also been demonstrated [21, 22]. Besides, studies have shown that SARS-CoV-2 spike protein has 10–20 times more affinity than the same protein in SARS-CoV [23]. Following ACE2 receptor and ligand (here spike protein) binding, structural modifications are made in the spike protein that lead to the integration of the envelope protein with the host cell membrane [24]. The virus’s RNA is then translated into the replica polyproteins pp1a and pp1b, which are further reduced to tiny proteins by virus-encoded proteinases. Coronavirus replication requires the replacement of the ribosomal framework during translation, and it creates both genomic copies and numerous copies of subgenomic RNA species via discontinuous transcription encoding the relevant viral proteins. Virion assembly is performed by interacting viral RNA and proteins in the endoplasmic reticulum (ER) and the Golgi complex, which are released as cell vesicles [21]. The pathological findings of patients with SARS-CoV-2 are very similar to those of patients with SARS-CoV and MERS-CoV. Reduced CD4 and CD8 levels, as well as enhanced HLA-DR and CD38 levels, are among the noteworthy outcomes of the blood samples. In lung cells, histopathological investigation of tissues from patients with SARS-CoV-2 revealed the virus’s cytopathic impact as well as signs of acute respiratory distress syndrome [16]. Lymphocytes are significantly reduced when MHC-like domains in the spike protein attach to NK and T cells (CD4^+^ and CD8^+^). In fact, E protein, by producing reactive oxygen species (ROS) kills the NK, CD4^+^ T, and CD8^+^ T cells. Liu Wenzhong and Li Hualan showed that S MHC-like structures in Delta, Alpha, Beta, and Lambda variants located in the N-terminal domain (NTD) at 127–194 and 144–162 in Delta and 62–80 in Alpha, Beta, and Lambda. In the Gamma variant, these structures are located in the S2 membrane fusion region at 616–676 and 1014–1114 [25].

## Primary SARS-CoV-2 in Wuhan

The pandemic began in 2019 in Wuhan, China, and led to the introduction of a new strain of coronavirus, hence the name Wuhan strain or wild-type SARS-CoV-2. The most important symptoms in patients were fever and shortness of breath [26]. Laboratory findings showed increased levels of IL-6, CRP, PCT, AST/ALT, bilirubin, ALP, GGT, LDH, ferritin, D-dimer, and neutrophils, and decreased levels of albumin and lymphocytes [26, 27]. It was reported that ground-glass opacity (GGO) with peripheral distribution is the prominent aspect of computerized tomography (CT) images in these patients which were unilateral and bilateral, and frequently one or two lobes are involved with particular signs [26, 28]. The main treatment protocols were antiviral drugs, antibiotics, glucocorticoids, recombinant intravenous immunoglobulin, and IFN-α2b [26, 29]. Ye et al. showed that convalescent plasma therapy is effective for patients with SARS-CoV-2 in Wuhan [30]. Lu et al. showed that the sequences of SARS-CoV-2 from several patients in Wuhan were 99.9% identical. They suggest that SARS-CoV-2 has a fast-spreading source [31]. The SARS-CoV-2 genome, as described in earlier sections, is highly similar in sequence and structure to other coronaviruses. In RNA viruses, genome mutations are a well-known evolutionary method. More than a thousand mutations have been found in the early SARS-CoV-2 genome, with 17 of them being high frequency [20].

## SARS-CoV-2 Alpha variant (lineage B.1.1.7)

The Alpha variant (lineage B.1.1.7) was discovered for the first time in November 2020 in the UK [32]. The Alpha variant is one of the concern variants that have 50% more transmissible character than wild-type SARS-CoV-2, and this variant became the dominant one in the USA and many European countries in the second quarter of 2021. The virus’s spike protein mutation was blamed for its high transmission [33]. Because it escapes antibody neutralization, this variation with receptor-binding domain (RBD) mutations causes hospitalizations and increases death rates. People with B.1.1.7 infection reported having a sore throat, fatigue, myalgia, and fever or a relative loss or alteration to their sense of smell or taste experience, while gastrointestinal symptoms, shortness of breath, and headaches were like those caused by wild type [34].

### Pathogenesis of Alpha variant

SARS-CoV-2 variant mutation in spike protein appeared in epidemiological and clinical aspects of the COVID-19 pandemic [35]. The spike mutations in the Alpha variant are consist of the substitution mutation in RBD, including N501Y, P681H, and deletion mutations in NTD at positions 69–70 and 144. Non-spike mutations contain nsp6:Δ106–108 and nucleocapsid mutations D3L, R203K, and G204R, which are probably responsible for transmission acceleration. S MHC-like 62–80 mutations in Alpha, Beta, and Lambda strains are considered an effective parameter for rapid spread [25]. The Alpha variant has the N501Y, K417N, E484K, and P681H mutations. N501Y is found in the Beta and Gamma VOCs in addition to the Alpha variant, which increases ACE2 affinity [35]. Furthermore, F490S and S494P occur in the receptor-binding motif (RBM) in several Alpha variations. Some mutations in the RBD, including E484K, F490S, and S494P, are related with reinfection and vaccination failure in Alpha variant sublineages [36].

## The SARS-CoV-2 Beta variant (B.1.351)

The Beta variant or lineage B.1.351 is one of the SARS-CoV-2 variants that, for the first time, was observed in South Africa in May 2020, which caused the second wave of infection in this country [37]. The US Centers for Disease Control and Prevention (CDC) announced that the Beta variant has shown a 50% faster transfer capability, and some evidence has proposed that some of the available vaccines are ineffective against this variant [38]. According to research on the Beta variant, a mutation in the spike protein may aid the virus in evading the immune response even in those who have been vaccinated [39]. The Beta lineage separation in the world was 33% in Europe, followed by Asia (25%), Africa (27%), the Americas (12%), and Oceania (3%). The Beta lineage after more than 1 year became the second-widest-spread lineage in the world in terms of its separation in more than 90 countries and regions [40]. Studies have suggested that young individuals with comorbidities are more susceptible to being affected by the Beta variant, and it can lead to more serious and more prevalent diseases compared with other variants [41].

### Pathogenesis of Beta variant

Studies have reported three significant mutations (K417N, E484K, N501Y) in the spike region of the Beta variant genome and also five (L18F, D80A, D215G, R246I, A701V) less important mutations in the spike. Moreover, for the spike region, four mutations (K1655N, SGF 3675–3677 deletion, P71L, and T205I) were reported [42]. The K417N/T mutation is existent in the Alpha, Beta (as K417N), and Gamma (as K417T) variant. The N501Y, K417N, and E484K mutation occurred in the RBD and RBM in the spike glycoprotein, so this mutation enables the virus to bind more easily to human cells [35]. Furthermore, E484K mutation was seen in the Alpha, Beta, and Gamma variants. Also, the Beta variant lacks the 69–70/del mutation observed in other variants [35].

## The SARS-CoV-2 Delta variant (B.1.617.2)

According to WHO data and reports from December 2020, India was home to one of the coronaviruses with the deadliest and most terrifying characteristics. This strain, known as the Delta variation (B.1.617.2), was significantly more pathogenic and quicker than its predecessors, since the spike protein was simpler to connect to the cell and prevent antibody binding [43, 44]. On the one hand, the Delta strain had a high ability to escape the immune system; on the other hand, at the time of emergence and spread of this strain, there was no collective safety, and these reasons caused the Delta strain to spread rapidly in many countries, including India, where 26% of the population was infected within 2 months [44, 45]. Despite the similarity of symptoms in the Delta and Alpha strains, those infected with the Delta variant suffered more severe symptoms and required intensive care and hospitalization in intensive care unit (ICU) wards [43, 46].

### Pathogenesis of Delta variant

Delta variant had 23 new mutations compared with the alpha strain, 12 of which involved the spike protein [47]. The spike protein allows the virus to attach to the surface of the host cell for entry. Two subunits of spike protein, S1 and S2, help bind ACE2 receptors and integrate in the host cell, respectively. On the other hand, spike protein is used to eradicate the virus by the host cell’s immune system because, after the virus enters the body and the immune system is exposed to the spike protein, it produces antibodies against it that will eradicate the virus [48]. The Delta (B.1.617.2) variant has eight substitutions (T19R, G142D, R158G, L452R, T478K, D614G, P681R, and D950N) in its spike protein and deletion at sites 156 and 157 [43]. The most important reason that has made the Delta variant such a highly contagious variant is the existence of new mutations, including T19R, L452R, T478K, D614G, and P681R, of which L452R and P681R are more important. Studies have shown that these mutations can increase the affinity of spike protein to bind to the ACE2 receptor [49]. Moreover, N: D63G and RdRp: G671S mutations of the Delta variant resulted in a high viral load [25]. According to previous research, the Delta form with the L452R mutation is able to escape from immune cells such CD8 T cells, allowing the virus to survive and continue to function in the host body [46]. Furthermore, since the E484Q mutation caused the virus to propagate quickly in Beta and Gamma species, the E484Q and L452R mutations were a more widespread issue [50, 51].

## SARS-CoV-2, Gamma variant (lineage P1)

The Gamma or P1 variant, also known as the Brazilian variant, was first reported in November 2020 in Brazil [52]. According to research, the Gamma variant is derived from B.1.1.28 [53]. Studies on the Gamma variant have shown that this species is 1.7–2.4 times more frequent than other species in Brazil. Besides, it was reported that people infected with other strains, including Alpha and Beta, previously are not immune to the Gamma variant [52]. The Gamma variant can produce reinfections; it is about 2.521- to 10-fold more transmissible than the wild type, highlighting it as the most transmissible variant among the identified SARS-CoV-2 VOCs [54, 55]. The frequency of Gamma variations rose by 55.6% from September 2020 to February 2021. On 17 January 2021, the first Gamma-infected patient was discovered in Sergipe. As a result, in late 2020 and early 2021, this coronavirus was the most frequent in Brazil. Symptoms in Gamma variations were similar to those in prior strains, although the intensity of symptoms was less [53]. Gonçalves et al. revealed that the P1 strain, related to transmissibility, higher virulence, and mortality rates, leads to more severe cases of SARS-CoV-2 in pregnant and postpartum women [56]. Moreover, there is some evidence of increased hospitalization and mortality among young people with the P1 variant in Brazil [57].

### Pathogenesis of Gamma variant

In general, the Gamma variant has 17 mutations. These mutations include L18F, T20N, P26S, D138Y, R190S, K417T, E484K, N501Y, D614G, H655Y, T1027I, and V1176F in the spike protein; S1188L, K179Q, E5665D in ORF1ab; N protein mutations including E92K in ORF8 and P80K; and one deletion in S6F3675-3677 in ORF1ab. After Omicron, which contains 15 mutations in the spike protein, the Gamma variant has the highest number of mutations (12 mutations) in the spike protein [47]. Dos Santos et al. reported that mutations in Gamma increase the transmission of this variant [53]. S MHC-like 616–676 and 1014–1114 region mutations were reasons for the higher mortality observed for P1 lineage infections [25], which contribute to the acceleration of transmission and higher infection rates that can reduce the antibody-mediated immunity, and this trait is attributed to some mutations in the RBD region (K417T, E484K, N501Y) of the S protein and D614G [58–61].

## SARS-CoV-2 Omicron variant (B.1.1.529)

On 26 November 2021, a new variant of SARS-CoV-2 was noted and called Omicron or B.1.1.529 (variant 21K and BA.1), first reported in South Africa [62, 63]. Omicron was first observed in Tshwane City, Gauteng Province, where, after becoming the dominant species, it accelerated and spread to other nearby areas [62]. Symptoms of the Omicron variant are similar to previous species, including Alpha (B.1.1.7), Beta (B.1.351), and Delta (B.1.617.2) [64]. Omicron is frequent in the younger age range, according to current research. Furthermore, persons infected with Omicron had a lower risk of hospitalization and fatality than people infected with earlier species [65–67]. On the other hand, other studies discovered that the Omicron form might increase mortality owing to mutations and antibody resistance [64].

### Pathogenesis of Omicron variant

This new member of the SARS-CoV-2 family (B.1.1.529) has an unusual number of changes and deletions of amino acids in its spike protein, especially in RBD. There are four regions in the RBD of the spike protein on which the available neutralizing antibodies act. All epitopes in Omicron’s N-terminal domain have mutations [63, 67]. According to the research, this strain has 59 mutations, 32 of which are connected to spike protein. There are 15 mutations in the RBD of Omicron spike protein, including S371L, S373P, S375F, K417N, N440K, G446S, S477N, T478K, E484A, Q493R, G496S, N501Y, and Y505H [68–70]. Besides, a del69/70 mutation in the S gene was revealed that is used for PCR detection of Omicron [67]. In general, the Omicron variant has three categories of unique mutations: the Q498R and N501Y mutations, which cause a stronger ACE2 binding; the H655Y, N679K, and P681H mutations, which increase the gap in S1/S2; and mutation in P203K and G204R in the N protein, which increases the virus load in infected patients [63]. Redd et al. revealed that, despite unique mutations in the spike protein of Omicron, most T-cell epitopes in the Omicron have not changed (CD8^+^ T-cell epitope in spike protein contains a single amino acid change), and infected patients can probably protect themselves through CD8^+^ T-cell responses against the Omicron variant [62, 63]. Besides, about ten mutations occurred during the interaction between ACE2 and RBD. According to Lupala et al., who used molecular dynamics simulation, RBD shows a stronger binding to ACE2 in the Omicron variant than in SARS-CoV-2 wild type [69]. Thus, it was suggested that B.1.1.529’s strong binding to ACE2 probably causes the higher prevalence [69, 70].

## The SARS-CoV-2 Lambda variant (lineage C.37)

WHO identified the SARS-CoV-2 lineage C.37 (Lambda variant) because of its aggressive spike protein mutation and high transmission rates in South American countries [71]. In December 2020, the Lambda variation was detected for the first time in Peru. Within 3 months, the Lambda variation was deemed a dominant variant since it was responsible for 80% of all cases. This increase in transmission was attributed to some mutations in this variant [72]. The Lambda variant is connected with other variants, especially the Delta variant [73]. This variant almost converted to the dominant variant in Peru, but in other parts of the world, including the UK and the USA, it did not exceed the prevalence of the Delta variant. It seems that the inconsistent prevalence of the Lambda variant is due to the “founder effect,” a critical factor in the pandemic [74]. In addition to common COVID-19 symptoms, the Lambda variant is associated with diarrhea, a symptom that had not been seen before, while there were no other gastrointestinal symptoms such as nausea or vomiting [75].

### Pathogenesis of Lambda variant

The Lambda variant has a seven-amino-acid deletion in the S gene, and the L452Q mutation is identical to the L452R mutations in the Delta and Epsilon variants [72]. The L452Q mutation and the D614G mutation together are responsible for its high transmission. Studies have suggested that the spike protein is the most important factor in its high transmission, and T76I and L452Q mutations are associated with the more infectious properties of the Lambda lineage [76]. The T859N mutation is the most important in Lambda, and it exists in B.1.526.1 (New York) is connected with monoclonal antibody neutralization reduction and convalescent and post-vaccination sera. Genome sequencing analysis revealed that the Lambda variant consists of 27 unique mutations (1 in ORF1a—deletion 3675–3677; 7 in the gene-encoding protein S—deletion 246–252, G75V, T76I, L452Q, F490S, D614G, and T859N; and 19 mutations frequent in other variants of the SARS-2 coronavirus) [77]. The mutation in the Lambda lineage occurred in the N gene (P13L, R203K, G204R, and G214C), ORF1a (T1246I, P2287S, F2387V, L3201P, T3255I, and G3278S), ORF1b gene (P314L and deletion of three amino acids SGF in positions 3675–3677), ORF9b gene (P10S), and S gene (G75V, T76I, R246N, a deletion of seven amino acids SYLTPGD in positions 247–253, L452Q, F490S, D614G, and T859N) [78]. The Lambda variant family contains a subvariant (PV29369) with 13 amino acid deletions, including camper to consensus Lambda, NTD substitutions G75V and T76I, and an additional E471Q substitution in the RBD, as well as one large NTD deletion (T63-G75) found only in Lambda C.37 with a 1% (GISAID) frequency. PV29369 and other Lambda subvariants have been co-circulating at low frequency. Furthermore, C.37 variant is a Lambda variant that contains an ORF1a gene (Δ3675–3677) deletion [79].

## SARS-CoV-2 Mu variant (B.1.621)

The Mu variant was first discovered in January 2021 in Colombia, and in August 2021 the WHO considered this variant a VOI and classified it as 21H or B.1.621 [80]. Mu was identified in 51 countries around the world (updated on 18 September 2021) [81]. WHO described B.1.621 as a lineage with the potential to escape the immune system. According to WHO assessments, the Mu variant is less clinically problematic than the other SARS-CoV-2 variants, but because of the common mutations in coronaviruses, there are concerns about contamination and resistance to drug efficacy and neutralization by the vaccine [82]. Comparative analysis of the critical spike mutations revealed that Mu shares biological similarities with the Beta strain regarding immune escape and infectivity [83].

### Pathogenesis of Mu variant

The Mu variant’s genome has numerous mutations in ORF1ab, ORF3a, ORF8, and spike genes, among others. With nine mutations, the spike protein has the most compared with other areas, including T95I, Y144T, and Y145S in the N-terminal domain; R346K, E484K, and N501Y in the RBD; and D614G, P681H, and D950N in the other regions. Furthermore, some of these mutations, including E484K in B.1.351 (Beta) and P.1, R346K in Omicron, P681H in Alpha, N501Y in Alpha and Beta, and D950N in Delta, are common in coronaviruses [84–86]. Xie et al. revealed that mutations in the spike protein facilitate virus entry into the cell via the ACE2 receptor; the transduction rate in the Mu variant is still lower than in the Delta variant [86]. Regarding the role of Mu variant mutations in escaping the immune system, as well as increasing the entry of the virus into the host cell, this variant could pose a new global challenge.

## Drug efficacy in different variants of SARS-CoV-2

The effectiveness of earlier medications in treating persons infected with new SARS-CoV-2 mutations is one of the most significant difficulties confronting researchers. Compared with the parent pandemic virus, the Alpha and Beta versions demonstrated lower sensitivity to numerous type I interferons [71]. Some type I interferons decrease sensitivity to Alpha variants by 112-fold compared with wild-type SARS-CoV-2. Studies have reported that F490S mutation results in a loss of susceptibility to bamlanivimab, although it is still susceptible to other FDA emergency use authorization (EUA)-approved monoclonal antibodies (mAbs). Furthermore, S494P showed more than tenfold and fivefold reduction when treated with bamlanivimab and casirivimab, respectively. Furthermore, E484K exhibits a three- to tenfold reduction in susceptibility to casirivimab and bamlanivimab and several other RBM class 1 and 2 mAbs [35, 87–89]. After the Beta outbreak, many studies were conducted to survey the efficacy of etesevimab and bamlanivimab and showed that these treatment strategies were not able to neutralize the B.1.351 (Beta) variant activity while they can still be effective against the B.1.1.7 (Alpha) variant and wild-type SARS-CoV-2. Moreover, the B.1.351 (Beta) variant can escape from neutralization induced by etesevimab and bamlanivimab, while the B.1.617.2 (Delta) variant could escape only from bamlanivimab [90]. Casirivimab and imdevimab, as two anti-RBD mAbs, can also neutralize both the B.1.351 (Beta) and B.1.617.2 (Delta) variants, though casirivimab’s IC_50_ increased markedly and indicates a reduction in its neutralization activity [91, 92]. The K417N/T mutation shows a sensitive reduction to etesevimab (> 100-fold) and casirivimab (tenfold) although it is still susceptible to bamlanivimab, imdevimab, and sotrovimab [93]. Another variant that has raised concerns is Gamma, which is thought to be resistant to antibodies owing to a mutation in the spike protein, particularly the E484K mutation [53]. According to studies on this version, Gamma is less resistant to antibodies than other variants [91].

In the Omicron variant, mAbs are designed on the basis of their effect on the spike protein to neutralize the effects of the virus in infected patients, so the mutation in the spike protein of Omicron variant may cause resistance to existing mAbs. Chen et al., using an artificial intelligence model, showed that the efficiency of the Eli Lilly mAb cocktail in the Omicron variant was reduced in terms of K417N, E484A, and Q493R mutations. Furthermore, E484A, Q493R, and Q498R mutations reduced the efficiency of the Celltrion Regdanvimab antibody, and the E484A mutation decreased the efficiency of Rockefeller University antibodies [70]. Furthermore, Petersen et al. observed that changes in the N-terminal domains may have resulted in resistance to current antibodies and boosted transmission power in their study [65]. Furthermore, studies have shown that all neutralizing antibodies certified by the WHO, with the exception of sotrovimab, have lost their anti-Omicron activity. In contrast to the N-terminal of the spike protein, the C-terminal of the spike protein is mutation-free. Since some present antibodies affect the C-terminal of spike protein (aa1148-1159, aa558-569, aa627-638), it can be hoped that some of the existing antibodies may affect Omicron [63]. However, the WHO has reported that drugs such as interleukin-6 (IL-6) receptor blockers and corticosteroids such as dexamethasone might be effective at improving the condition of a patient infected with Omicron [64].

Moreover Lambda variant drug sensitivity was investigated. Data showed that the decreases in neutralization to monoclonal antibodies and neutralizing antibodies originated from L452Q and F490S mutations. Thus, current vaccines may not afford good protection against the Lambda variant. Bamlanivimab may have a reducing effect on binding affinity to the Delta variant, while this therapeutic antibody in the Lambda variant has reduced efficacy owing to Lambda’s L452R mutation [73]. The Lambda variant spike protein contains a novel deletion (Δ246-252) and some nonsynonymous mutations (L452Q, F490S, G75V, T76I, T859N, and D614G). Furthermore, there are multiple nonsynonymous mutations and a new deletion in the spike genes (e.g., Δ246-252, G75V, T76I, L452Q, F490S, D614G, and T859N). Only the Lambda variation has the RBD mutations L452Q and F490S, as well as the NTD deletion Δ246–252. Moreover, two alterations in the RBD zone, namely F490S and L452Q, are responsible for the decrease in antibody neutralization [47].

On the other hand, the E484K, N501Y, and D614G mutations in the Mu variant are associated with its lower sensitivity to antibodies both in patients with SARS-CoV-2 and in vaccinated people. Moreover, the efficiency and connection between mutations in Mu spike protein and neutralization resistance were measured by comparing neutralization efficacy against the D614G, Mu, and Delta spike pseudotyped viruses, and it was revealed that the neutralizing potency of the serum was significantly reduced 2.2- and 1.5-fold for the Mu variant and the Delta variant, respectively, compared with activity against D614G. These results revealed that the Mu strain has an unanticipated eminent neutralizing resistance to inactivated vaccine-elicited antibodies [86]. Also, a recent study revealed that the BNT162b2 mRNA vaccine in patients with the Beta variant and natural infection with SARS-CoV-2 led to significant resistance in patients with the Mu variant [83].

## Vaccine effectiveness against SARS-CoV-2 variants of concern

The administration of COVID-19 vaccinations provides a significant advantage in terms of slowing the spread of COVID-19 [94]. According to a WHO report, there are 351 candidate vaccines as of 18 January 2022, with 154 in clinical trials [95]. Many prospective vaccines against SARS-CoV-2 are based on different platforms, such as mRNA-based vaccines, viral-vectored vaccines, inactivated virus-based vaccines, and recombinant proteins [96]. Several vaccine candidates have completed phase III clinical studies and are reported to be effective in immunizing against COVID-19 after their rollout via EUA. Vaccine candidates Oxford–AstraZeneca, Pfizer–BioNTech BNT162, Moderna’s mRNA-1273, Sinovac’s CoronaVac, Johnson & Johnson, Sputnik-V, and Sinopharm are leading the race [97, 98]. Pfizer–BioNTech and Moderna contain a genetic component of the SARS-CoV-2 virus that causes COVID-19. Genetic material, RNA in the case of Pfizer–BioNTech and Moderna, code for a specific virus protein. One of the most searched viral vectors is adenovirus, presently used by Oxford–AstraZeneca. Adenoviruses are common cold viruses with a double‐stranded DNA genome. This type of vaccine uses an unrelated harmless virus (the viral vector) to deliver SARS-CoV-2 genetic material [99]. Inactivated vaccines like Sinopharm BBIBP-CorV contain the killed SARS-CoV-2 virus, which is recognized by the immune system to initiate a reaction without causing COVID-19 [97].Recently, several variants of concern have emerged, including Alpha (B.1.1.7), Beta (B.1.351), Gamma (P.1), Delta (B.1.617.2), and Omicron (B.1.1.529), which are associated with increased transmissibility and virulence [6]. All COVID-19 vaccines had a high efficacy against the original strain and the variants of concern. At the moment, 60.9% of the worldwide population has received at least one dose of a COVID-19 vaccination [100]. Two doses of SARS-CoV-2 vaccination are extremely successful in avoiding hospitalization, severe cases, and fatalities caused by COVID-19, although the vaccine effectiveness of different groups of days after the second vaccine dose reveals no statistically significant changes [101]. The vaccine’s efficacy against SARS-CoV-2 infection is 89.1% in fully vaccinated people [102]. It has been reported that the pooled vaccine effectiveness is 85–87.5% for the protection against the Alpha variant of SARS-CoV-2 infections, 56.5–75% for the Beta variant (Table 1), 54% for the Gamma variant, 74–80.1% for the Delta variant, and 88.0% for the Omicron variant [103, 104].

**Table 1:** The efficacy of vaccines against SARS-CoV-2 variants of concern

Vaccine	Manufacturer	Type of vaccine	Vaccination status	Efficacy against Alpha	Efficacy against Beta	Efficacy against Gamma	Efficacy against Delta	Efficacy against Omicron
BNT162b2	Pfizer–BioNtech	RNA-based	Single dose	29.5% [105] 70% [106] 46% [107]	16.9% [105] 43% [108] 54.7% [109]	61.0	30.7% [110] 56% [111] 64.2% [112]	55.2% [113]
			Double dose	89.5 [105] 85% [106], 92% [107] 95.3% [114] 94% [110]	75.0% [105], 77% [115] 88% [108] 84.8% [109]	–	88.0% [110] 87% [111] 75% [116] 91% [117] 53.5% [112]	88.0% [118]
mRNA-1273	Moderna	RNA-based	Single dose	88.1 [119], 54.5% [120] 67.0 [121]	61.3% [119]	61% [121]	72% [111] 79.0% [112]	36.7% [113]
			Double dose	100% [119] 84.4% [120]	96.4% [119]	–	91% [117] 84.8% [112] 87.9% [122]	30.4% [123]
ChAdOx1 nCoV-19	AstraZeneca	Viral vector	Single dose	48.7% [110], 60% [124], 64% [111], 68%[125], 62% [126], 95% [127]	75.4% [128]	33.4% [129]	30.7% [110] 67% [111] 46.2% [130]	–
			Double dose	74.5% [110], 70.4% [131]	21.9% [128]	77.9% [129]	67.0% [110]	86·4% [132]
BBIBP‐CorV	Sinopharm	Inactivated virus	Single dose	–	–	–		
			Double dose	–	–	–		–
CoronaVac	Sinovac	Inactivated virus	Single dose	–	–	49.6% [133], 12.5% [134]		–
			Double dose	–	–	36.8% [133], 46.8% [134] 68.1% [135]	59.0% [135]	–
NVX‐CoV‐2373	Novavax and CEPI		Single dose	85.6%		–		–
			Double dose	60.1% [136]	–	–	–	–

## Conclusion

COVID-19 is a global crisis that has killed many people. Overcoming this virus is a major challenge. The emergence of various mutations in the genome of this virus, which has resulted in the production of new strains of the virus, is the fundamental difficulty in managing and stopping the pandemic. The most significant of these alterations occurs in the spike gene, which enhances affinity for the spike protein and the ACE2 receptor, resulting in increased incidence and toxicity of new strains. Furthermore, mutations in the virus’s genome have altered the response to drugs used to treat patients with different strains of SARS-CoV-2 infection. On the other hand, vaccination has been shown to significantly reduce acute disease as well as mortality; however, the development of new strains has changed the effectiveness of different vaccines.

## Data Availability

Not applicable.

## Abbreviations

ACE2: Angiotensin-converting enzyme 2
α-CoV: Alphacoronovirus
β-CoV: Betacoronavirus
δ-CoV: Deltacoronavirus
γ-CoV: Gammacoronavirus
MERS-CoV: Middle East Respiratory syndrome coronavirus
nsps: Nonstructural proteins
ORFs: Open reading frames
RBD: Receptor-binding domain
RBM: Receptor-binding motif
SARS-CoV-2: Severe acute respiratory syndrome coronavirus 2
TRS: Transcription regulatory sequences
UTRs: Untranslated regions
VOC: Variants of concern
VOI: Variants of interest
WHO: World Health Organization
